# Signaling pathway switch in breast cancer

**DOI:** 10.1186/1475-2867-13-66

**Published:** 2013-06-27

**Authors:** Arnaud Guille, Max Chaffanet, Daniel Birnbaum

**Affiliations:** 1 Centre de Recherche en Cancérologie de Marseille, Oncologie Moléculaire, “Equipe labellisée Ligue Contre le Cancer”, UMR1068 Inserm, CNRS UMR7258, Institut Paoli-Calmettes, Aix-Marseille Université, 27 bd. Leï Roure, BP 30059, Marseille 13273, France

**Keywords:** Next generation sequencing, Breast cancer, Signaling pathways, Cell cycle, Kinases

## Abstract

Next generation sequencing studies have drawn the general landscape of breast cancers and identified hundreds of new, actual therapeutic targets. Two major signaling pathways seem to be altered in a vast proportion of breast cancers. The PI3 kinase/AKT pathway is activated and the JUN/MAPK pathway is repressed. Via the regulation of the cell cycle this metabolic switch impacts on the balance between self-renewal, proliferation and differentiation of the tumor-initiating cells

## Background

Recent results from next generation sequencing (NGS) studies have established the repertoire of driver gene mutations and copy number alterations (CNA) in breast cancer [1-4]. Nearly 900 cancers representative of all major expression subtypes (basal, luminal A and B, ERBB2 and normal-like) have been studied. Many recurrent mutations have been uncovered. Mutations in *TP53, PIK3CA, GATA3* and *PTEN* genes are among the most frequent. These studies have forever changed our understanding of mammary oncogenesis.

## Hypothesis

Many studies will extend these pioneering ones but it is already possible to speculate further on the NGS data. Data analysis revealed that some alterations (CNA and/or mutations) never occur in the same tumor, i.e. are mutually exclusive [1-4]. Two main signaling pathways seem to be targeted, the PI3K/AKT pathway and the JUN/MAPK pathway [1-4]. Alterations in components of the PI3K/AKT pathway (PIK3CA, PIK3R1, AKTs, PTEN, INPP4B…) are mutually exclusive but strikingly, amplification and upregulation of genes encoding receptor-type tyrosine kinases (RTKs) (IGF1R, EGFR, ERBB2) are also (globally) mutually exclusive with alterations of the PI3K/AKT pathway. This suggests that the primary role of RTK amplification or mutation is to activate the PI3K/AKT pathway. Thus, in the normal mammary epithelium these RTKs are repressed or expressed at a low level and their signaling is primarily oriented toward the JUN/MAPK pathway, whereas when upregulated in tumor cells they stimulate the PI3K/AKT pathway. To obtain this dosage effect could be the reason for the amplification of *ERBB2* and *FGFR1* genes, although there could be other reasons [5]. It is known that the PI3K/AKT pathway is activated in tumors with mutated EGFR or overexpression of ERBB2 and determines the response to ERBB targeted inhibitors [6]. Within the JUN/MAPK pathway alterations of the components are also mutually exclusive [1]. Components of the JUN/MAPK pathway are inactivated by deletions and mutations, such as *MAP2K4* and *MAP3K1*, or by amplifications, such as *PAK1*. Most importantly, alterations leading to the activation of the PI3K/AKT pathway and those leading to the inactivation of the JUN/MAPK pathway are mutually exclusive [1]. Finally, not only mutations and genomic rearrangements affect genes encoding components of the two pathways but opposite modifications in expression patterns of these genes could also participate to their switch in breast cancer.

Our hypothesis is that one important consequence of mutations, CNA and modifications of expression is to shift cell signaling in the targeted mammary epithelial cell from an active “JUN/MAPK pathway – inactive PI3K/AKT pathway” state to an active “PI3K/AKT pathway – inactive JUN/MAPK pathway” state.

### Testing the hypothesis

**Figure 1:**
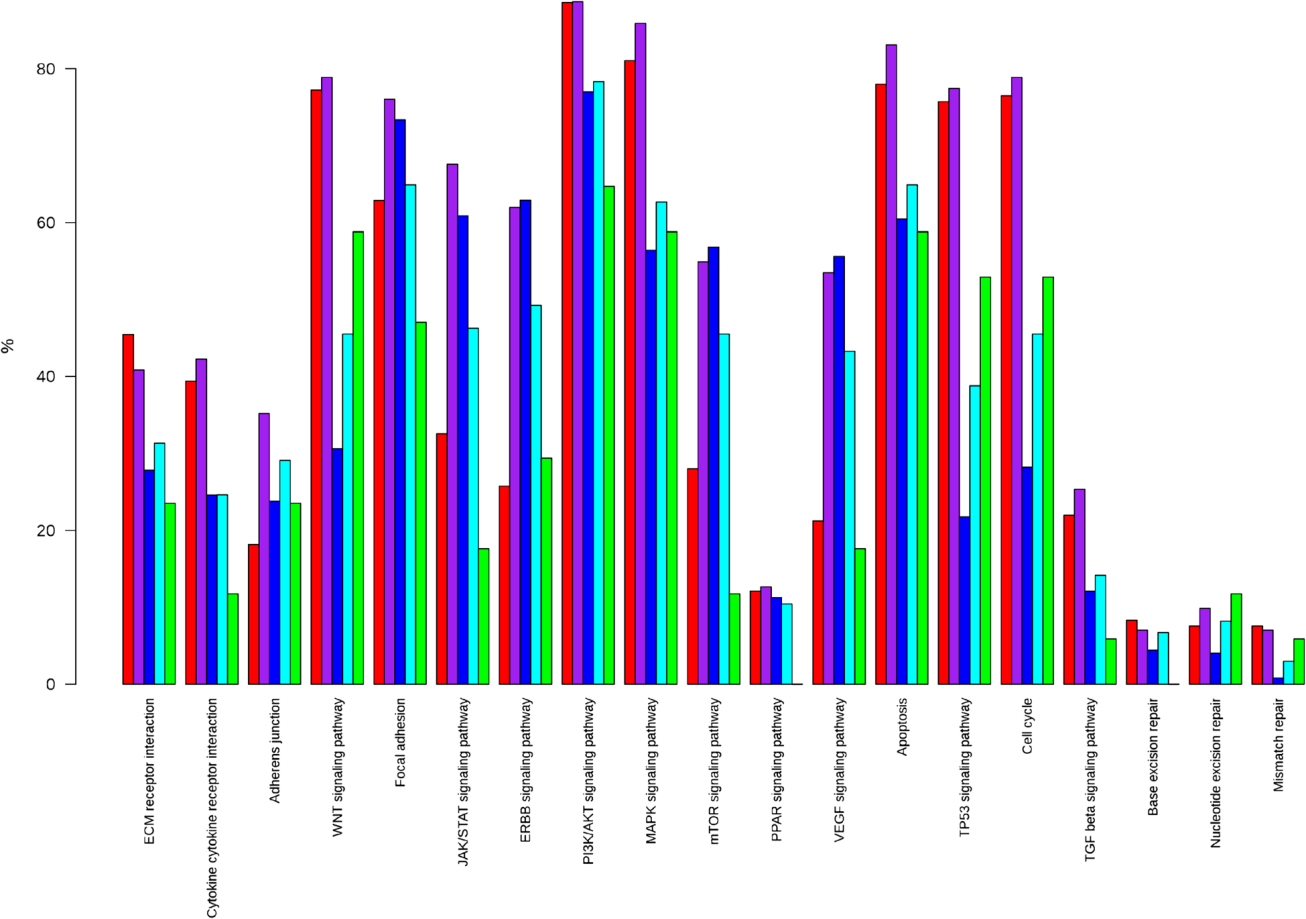
**Altered KEGG pathways in breast cancers.** In the bar-plot depicting the proportion of cancer samples with altered pathways (19 pathways are listed in the x-axis) the y-axis represents the percentage of samples altered in a given pathway stratified by subtype (red for basal, purple for ERBB2, blue for luminal A, cyan for luminal B, and green for normal-like). The analysis was done in 602 samples from different studies in which the molecular subtype (using PAM50) was available (466 from ref. 1, 98 from ref. 2, 38 from ref. 3).

**Figure 2:**
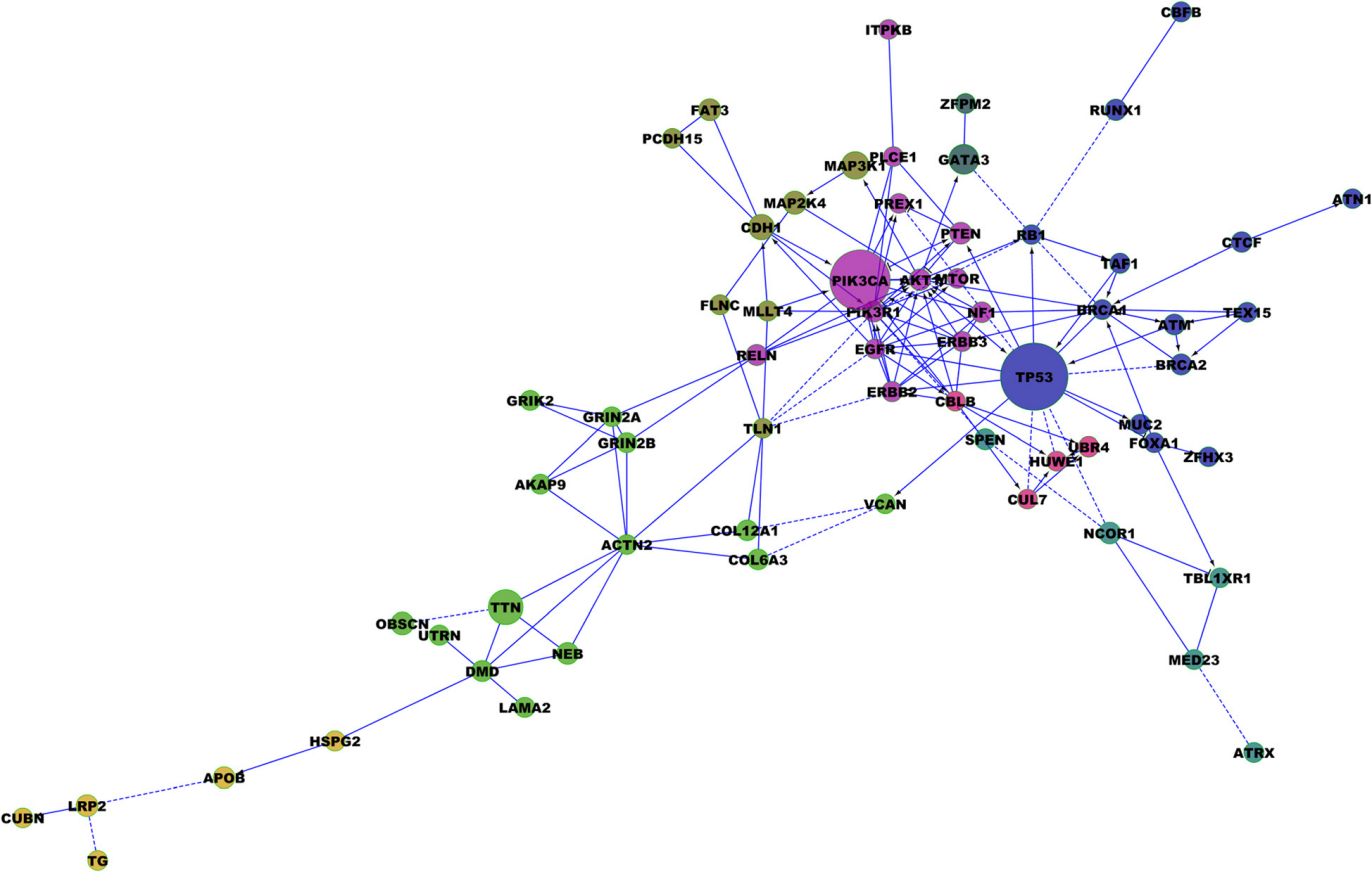
**Subnetwork representation of protein interactions based on 78 mutated genes in 602 breast cancers **[1]**-**[3]**.** Gene networks were inferred using the Reactome FI Cytoscape Plugin [7]. A total of 182 genes with more than 7 mutations in 602 breast cancer samples were used. Among them, 78 (43%) were mapped in the subnetwork and subsequently clustered into 12 modules, six of which were greater than 4. Finally, modules functions were assessed with pathway enrichment analysis (FDR < 0.05). Three main pathways can be recognized: the P53 pathway and DNA repair (14 genes/18%) in blue, the PI3K/AKT signaling pathway (13 genes/18%) in pink and the MAPK pathway (8 genes/10%) in green.

In the diagram shown in Figure 1 we have represented 19 KEGG metabolic and signaling pathways and cell processes altered by mutations in their components in the 602 breast cancer samples of the NGS studies for which the molecular subtype had been assigned [1-4]. All pathways and processes are altered, although to different extents. Many potential therapeutical targets can be found in these pathways. Two main signaling pathways are targeted (Figure 2), the JUN/MAPK pathway and the PI3K/AKT pathway [1-4]. Mutations in *PTEN*, amplifications and mutations in *PIK3CA* and *AKT* genes, the *MAGI3-AKT3* gene fusion [3], activate the PI3K/AKT pathway whereas mutations in *MAP3K1, MAP3K13* and *MAP2K4* inactivate the JUN/MAPK pathway. The PI3K/AKT and JUN/MAPK pathways are intimately related and intricate. For example, AKT activation inhibits MAP2K4. This interaction and the mirror effect of the alterations on the two signaling pathways suggest that the PI3K/AKT pathway stimulates the growth of tumor cells whereas the JUN/MAPK pathway has an opposite effect and that the two pathways are the two sides of the same coin.

**Figure 3:**
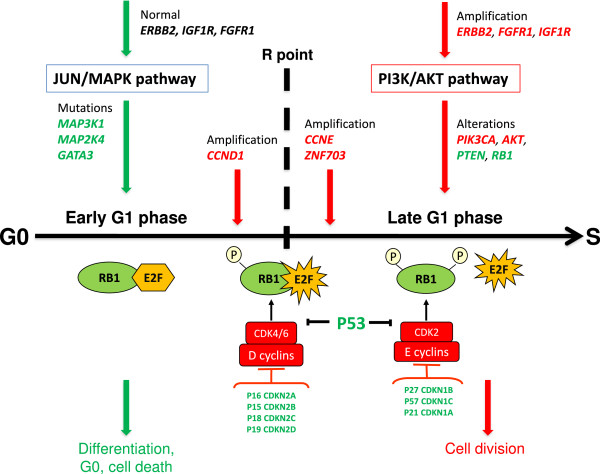
**Hypothetical representation of opposite downstream effects of two signaling pathways on the G1 phase of the cell cycle in a breast tumor**-**initiating cell.** During the early G1 subphase the cell integrates signals that may lead it to several fates (differentiation, apoptosis, senescence, quiescence, pause for DNA repair, progression to S phase) [8]. Inhibition of the JUN/MAPK pathway shortens the early G1 subphase rendering the cell independent of differentiation and apoptotic factors. In the same time, activation of the PI3K/AKT pathway induces the cell to progress to late G1 subphase beyond the restriction (R) point; the cell may then undergo symmetric (proliferation) or asymmetric (self-renewal) division. In green: proteins with repressive effect, in red: proteins with activating effect.

The downstream effects of the activated PI3K/AKT and inhibited JUN/MAPK pathways are multiple, but at least two could be of primary importance for the behavior of the tumor-initiating cell that fuels the tumor growth. A first major effect could be on the cell cycle. During the G1 phase of the cell cycle, a checkpoint before entering S phase, also called the restriction (R) point, has been defined as an important cell cycle stage controling various cell fates [8] (Figure 3). The G1 phase of the cell cycle has been thus divided into an early, signaling factor-dependent subphase, controled by D cyclins and a late, factor-independent subphase, controled by E cyclins and a fully inactivated (hyperphosphorylated) RB protein. The JUN/MAPK signaling pathway plays a role in the early G1 subphase, where the cell may be induced into quiescence, senescence or committed to differentiation, depending on the presence of external factors [9]. When entering the late G1 subphase, quiescence, cell death or differentiation are no longer options and the cell progresses to S phase and then to either symetric (proliferation) or asymetric (self-renewal) division. At the level of the tumor-initiating cell, the PI3K/AKT pathway could stimulate self-renewal and/or proliferation and the JUN/MAPK pathway cell differentiation or cell cycle arrest. A compromised JUN/MAPK pathway and constitutively activated PI3K/AKT pathway could force the cell to go through the R point and progress to S-phase. In this scheme, amplification of cyclin D1 appears as a particularity; however, it occurs mainly in luminal tumors, which are the most differentiated and less proliferative of all breast tumors. Combined cyclin D1 amplification (or a mutation with similar effect) and PI3K/AKT pathway would give luminal tumors their particular phenotype. In addition to the signaling pathways, the P53 protein acts as an important checkpoint on the way to the late G1 subphase. Inactivation of P53 (by way of mutations or deletions of the *TP53* gene, or another mechanism such as the amplification of the *MDM2* gene) is a prominent feature of all subtypes of breast cancer. However, it is more frequent in aggressive breast cancers, especially the basal cases, where alterations of the PI3K/AKT and JUN/MAPK pathways are less predominant. Basal breast cancers display a rearranged genome. P53 inactivation, which has consequences on the stability of the genome beyond those that could result from an activation of signaling pathways, could be the major cause of the basal phenotype.

A second major effect of PI3K/AKT pathway activation and JUN/MAPK inhibition is on the metabolism of the tumor-initiating cells. The latter are particularly resistant to oxidative processes and ROS production. The PI3K/AKT pathway, beside its role on cell growth and survival, regulates a number of metabolic processes including cell glucose uptake and neoglucogenesis in relation with nutrient availability and redox and energy conditions. It plays an important role in the glycolytic phenotype of tumors [10]. Forkhead transcription factors FOXO are downstream of the PI3K/AKT and JUN/MAPK cascades. FOXOs play a major role in the regulation of both the cell cycle and metabolism [11]. They prevent stem cells to accumulate ROS and allow DNA repair or quiescence. FOXOs are activated by the JUN kinase signaling pathway in the presence of oxidative stress. Conversely, activation of the PI3K/AKT and inhibition of JUN/MAPK pathways overrules the tumor suppressive effect of FOXOs and allows G1 progression. With respect to cell cycle and cell fate decisions FOXO transcription factors could thus represent important integrators of the two signaling pathways that are predominantly altered in breast cancer. They are therefore important targets in mammary oncogenesis. *FOXO* genes have not been found among the most frequently altered genes in breast cancer in NGS studies [1-4]; they are probably not directly targeted (yet, we have recently found rare mutations and deletions of the *FOXO3* gene; Cornen et al., submitted). FOXOs functions may also be modified but maintained to ensure some degree of metabolic regulation in the tumor-initiating cells. FOXOs and P53 act on the same cellular processes and transcription programs, and are intimately cross-regulated [11].

### Implications of the hypothesis

The NGS studies have drawn the general landscape of breast cancers and identified hundreds of new, actual targets. Although the role of many of them needs clarification, especially regarding the transcription factors (e.g. GATA3, RUNX, NCORs…), two major signaling pathways seem to be altered. This is good news since strategies to counter the consequences of pathway alterations are known and could be improved. Inhibiting the PI3K/AKT pathway appears as a paramount strategy to stop tumor growth [12]. If it fails one could adopt a strategy that takes into account both the PI3K/AKT and JUN/MAPK pathways but because they are truly interrelated this may not be helpful. Targeting additional alterations or pathways (including synthetic lethal ones) [13] outside the PI3K/AKT and JUN/MAPK pathways could be more successful.

## Competing interests

The authors declare that they have no competing interests.

## Authors’ contributions

The hypothesis came to view during discussions between the three authors. All authors read and approved the final manuscript.
